# *Pseudomonas aeruginosa* in Cancer Therapy: Current Knowledge, Challenges and Future Perspectives

**DOI:** 10.3389/fonc.2022.891187

**Published:** 2022-04-28

**Authors:** Zheng Pang, Meng-Di Gu, Tong Tang

**Affiliations:** ^1^ Innovative Institute of Chinese Medicine and Pharmacy, Shandong University of Traditional Chinese Medicine, Jinan, China; ^2^ School of Art & Design, Qilu University of Technology (Shandong Academy of Sciences), Jinan, China

**Keywords:** *Pseudomonas aeruginosa*, cancer therapy, anti-cancer agent, vector, immunotoxin

## Abstract

Drug resistance, undesirable toxicity and lack of selectivity are the major challenges of conventional cancer therapies, which cause poor clinical outcomes and high mortality in many cancer patients. Development of alternative cancer therapeutics are highly required for the patients who are resistant to the conventional cancer therapies, including radiotherapy and chemotherapy. The success of a new cancer therapy depends on its high specificity to cancer cells and low toxicity to normal cells. Utilization of bacteria has emerged as a promising strategy for cancer treatment. Attenuated or genetically modified bacteria were used to inhibit tumor growth, modulate host immunity, or deliver anti-tumor agents. The bacteria-derived immunotoxins were capable of destructing tumors with high specificity. These bacteria-based strategies for cancer treatment have shown potent anti-tumor effects both *in vivo* and *in vitro*, and some of them have proceeded to clinical trials. *Pseudomonas aeruginosa*, a Gram-negative bacterial pathogen, is one of the common bacteria used in development of bacteria-based cancer therapy, particularly known for the *Pseudomonas* exotoxin A-based immunotoxins, which have shown remarkable anti-tumor efficacy and specificity. This review concisely summarizes the current knowledge regarding the utilization of *P. aeruginosa* in cancer treatment, and discusses the challenges and future perspectives of the *P. aeruginosa*-based therapeutic strategies.

## Introduction

Cancer is one of the most dreaded diseases of human, and is the first or second leading cause of death in most countries of the world (1). The hallmark of cancer includes uncontrolled proliferation, resistance to cell death, insensitivity to growth suppressors, sustained angiogenesis, replicative immortality, and abilities of invasion and metastasis (2). The conventional treatment of cancer includes surgery, radiotherapy and chemotherapy, which are well-established and effective in eliminating fast-growing cancer cells (3). However, these conventional cancer therapies have a lot of limitations, including inefficacy in drug-resistant tumors, lack of tumor specificity, undesirable cytotoxicity to normal cells and adverse effects on cancer patients (4). In the past decade, alternative and complementary cancer therapies including nanoparticles, extracellular vesicles for delivering therapeutic agents, gene therapy, targeted therapy, diet therapy, herbal medicine, bacteriotherapy and magnetic hyperthermia have gained a high degree of research attention, and exhibited excellent anti-tumor effects *in vitro* and in animal models (5, 6). However, most of the therapeutic approaches are currently under preclinical and clinical investigation.

Bacteria-mediated cancer therapy has emerged as a promising approach in cancer treatment, which is capable of overcoming some of the limitations of conventional cancer therapies (7). Many obligate or facultative anaerobic bacterial species including *Clostridium* sp., *Bifidobacterium* sp., *Salmonella* sp., *Bacillus* sp., *Escherichia coli*, *Listeria monocytogenes* and *Pseudomonas aeruginosa* have been reported to penetrate and replicate in the hypoxic regions of tumors or accumulate in the tumor microenvironment (8, 9). Furthermore, these therapeutic bacteria are able to inhibit tumor growth and metastasis by production of toxins and stimulation of host immune responses (10). In addition, bacteria can be genetically engineered for their accessible genes, and used as vectors to deliver anti-tumor agents or immunomodulatory proteins to tumor sites (11, 12). Importantly, the genetically modified, live attenuated bacteria can be eliminated by antibiotics or triggering and strengthening host immune responses by immunomodulators such as cytokines and host defense peptides after the cancer treatment to prevent unintended infections (13–15).

**Figure 1:**
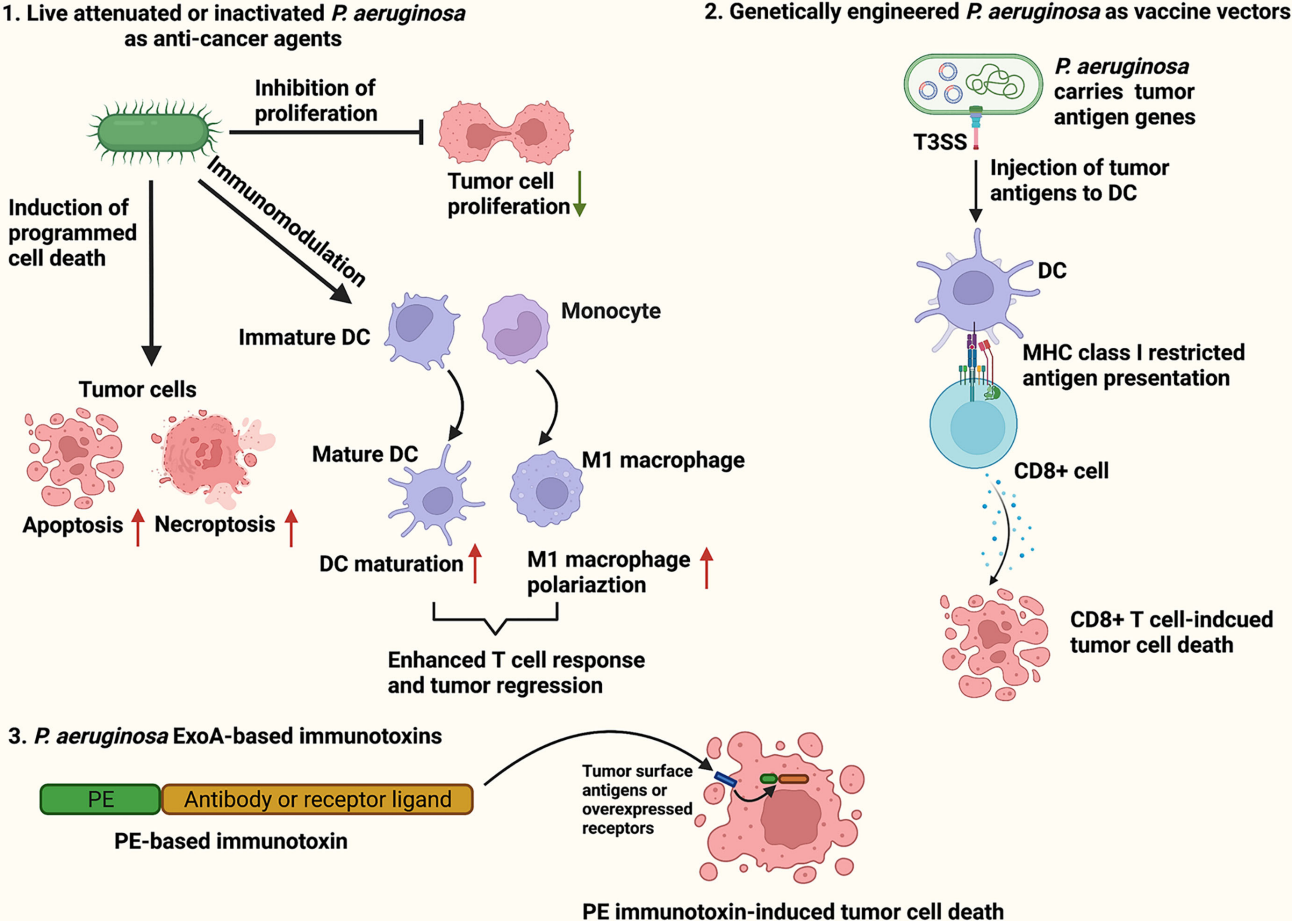
Schematic illustration of *P. aeruginosa*-based cancer therapies. The *P. aeruginosa*-based therapeutic strategies for cancer treatment include live attenuated or inactivated *P. aeruginosa* as anti-cancer agents (1), *P. aeruginosa* as vaccine vectors for tumor antigen delivery (2), and *P. aeruginosa* ExoA-based immunotoxins (3).

*Pseudomonas aeruginosa* is a Gram-negative, aerobic bacteria that is harmless to healthy individuals but causes severe infections in cystic fibrosis patients and immunocompromised individuals (16). Although *P. aeruginosa* is categorized as an aerobe, it acts as a facultative anaerobe capable of using alternative electron acceptors such as nitrate (NO^3−^), nitrite (NO^2−^) and nitrous oxide (N_2_O) to produce energy under oxygen-limited conditions (17). Live attenuated, inactivated or genetically modified *P. aeruginosa* have been reported to effectively cause tumor regression in mouse models by inducing cancer cells to undergo programmed cancer cell death (18–21), dampening proliferative signaling (22–24), and activating host anti-tumor responses (25, 26). Furthermore, many *P. aeruginosa* virulence factors including exotoxin A (ExoA), exoenzyme T (ExoT), azurin, cyclodipeptides, Pa-caspase recruitment domain (Pa-CARD) and rhamnolipids have been found to exert potent cytotoxicity against various cancer cells (27–32). In particular, ExoA is the most toxic virulence factor of *P. aeruginosa*, and widely applied in construction of immunotoxins for targeted cancer therapy (33). The present review aimed to concisely summarize and discuss the current findings on *P. aeruginosa*-based cancer therapeutic approaches, including live attenuated or inactivated *P. aeruginosa* as anti-cancer agents, *P. aeruginosa* as vaccine vectors for tumor antigen delivery, and *P. aeruginosa* ExoA-based immunotoxins (**Figure 1**).

## Live Attenuated or Inactivated *P. aeruginosa* as Anti-Cancer Agents

The role of bacteria as anti-cancer agents was first identified by German physicians W. Busch and F. Fehleisen who observed tumor regression in the cancer patients suffered from erysipelas caused by *Streptococcus pyogenes* infection (34). In 1891, William Coley, an American surgeon, inoculated cancer patients with *S. pyogenes*, which was the first time that bacteria were used to treat cancer (35). Hypoxia is a common feature of solid tumors, which is characterized by insufficient oxygen supply caused by rapid tumor growth. (36). Moreover, the hypoxic tumor microenvironment promotes tumor growth and angiogenesis (37). A number of obligate anaerobes including *Clostridium* sp. and *Bifidobacterium* sp. and facultative anaerobes including *Salmonella* sp., *Bacillus* sp., *E. coli*, *L. monocytogenes* and *P. aeruginosa* have been reported to colonize and replicate in the hypoxic region of tumors (10). Of note, the bacteria used in cancer treatment are required to be attenuated or genetically modified to reduce their toxicity and the ability to replicate before applying in treatment.

*Pseudomonas aeruginosa*-mannose sensitive hemagglutinin (PA-MSHA) is a genetically engineered *P. aeruginosa* strain characterized by high expression of mannose-sensitive hemagglutination (MSHA) fimbriae on its surface, which lowers toxicity by minimizing the exposure of other surface virulence factors such as LPS and flagella (38). Moreover, the MSHA fimbriae has been recognized as a novel ligand of Toll-like receptor 4 (TLR4) (39). Previous studies have shown that PA-MSHA suppressed tumor progression by induction of apoptosis through activating caspase-3, -8 or -9 (18, 19, 40), inhibition of cancer proliferative signaling such as EGFR, NRF2/KEAP1 and hedgehog signaling (22–24), and modulation of host immune responses through enhancing T cell responses, dendritic cell (DC) maturation and M1 macrophage polarization (25, 26, 41). Generally, the live or inactivated PA-MSHA was administered by subcutaneous injection in clinical trials or mouse models, which enhanced host anti-tumor immune responses systemically (19, 26, 42, 43). Moreover, the live PA-MSHA may enter the tumor tissues from blood circulation *via* passive entrapment in the leaky tumor vasculature or chemotaxis toward the chemicals released by the dying tumor tissue (44, 45). The heat-inactivated PA-MSHA combined with chemotherapy has been applied in clinical trials for treatment of breast cancer, lung cancer and lymphoma in China (42, 43, 46, 47). Most of the clinical studies suggested that the combination of inactivated PA-MSHA and chemotherapy drugs could improve the clinical efficacy of chemotherapy without increasing toxicity to cancer patients. Furthermore, the patients who were more responsive to PA-MSHA stimulation may receive better treatment outcomes. Lv et al. carried out a phase II clinical trial of inactivated PA-MSHA combined with capecitabine for treatment of HER2-negative metastatic breast cancer, and found that the patients with moderate immune-related adverse events (IRAEs) such as fever or skin induration caused by PA-MSHA injection manifested higher survival (25.4 months vs. 16.4 months) and longer progression-free survival (8.2 months vs. 3.1 months) compared to the patients who had no or mild IRAEs (43). One of the challenges for *P. aeruginosa*-based cancer treatment is that *P. aeruginosa* is able to induce a self-degradative and recycling process termed autophagy (48–50), which increases the resistance of cancer cells to chemotherapy and radiotherapy (51, 52). Xu et al. identified that PA-MSHA induced autophagy in human breast cancer cells through upregulation the endoplasmic reticulum (ER) stress-activated IRE1 signaling, and treatment of an autophagy inhibitor 3-methyladenosine (3-MA) enhanced the PA-MSHA-induced apoptosis of breast cancer cells *in vitro* and tumor regression *in vivo* (53). This study suggests that inhibition of autophagy can increase the effectiveness of *P. aeruginosa*-induced tumor regression.

In addition to PA-MSHA, the anti-tumor effects of a clinical isolate of *P. aeruginosa* strain 1409 were examined *in vitro* and *in vivo* by Qi et al. (20). The authors demonstrated that *P. aeruginosa* 1409 induced a programmed necrosis (necroptosis) of TC-1 tumor cells through activation of TLR4-RIP3-MLKL, and the HMGB1 released by the dying tumor cells further induced DC maturation and migration to tumor sites. Subsequently, the mature DC promoted T-cell responses by presenting tumor-associated antigens, thus resulting in remarkable tumor suppression in a TC-1 grafted tumor mouse model (20). This study indicates that the pathogenic clinical strains of *P. aeruginosa* could induce a potent anti-tumor response by reshaping tumor microenvironment. However, the live pathogenic *P. aeruginosa* strains must be attenuated or modified to reduce toxicity prior to clinical use due to the weakened immune system of cancer patients, which increases the prevalence of *P. aeruginosa* infections (54). Furthermore, this bacterial pathogen is resistant to many of the currently available antibiotics such as aminoglycosides, quinolones and β-lactams (55). Thus, clearance of the pathogenic *P. aeruginosa* strains after treatment is more difficult compared to other therapeutic bacteria.

## *Pseudomonas aeruginosa* as Vaccine Vectors for Tumor Antigen Delivery

Many anaerobic bacteria are recognized as the attractive vectors for the delivery of therapeutic genes to tumors for their ability to internalize and replicate inside tumor cells or grow in the hypoxic tumor microenvironment (56). The therapeutic genes encode anti-tumor agents, cytotoxic peptides, therapeutic molecules or prodrug-converting enzymes (57). The ideal bacterial vectors would be administered systemically, and selectively deliver the therapeutic genes to tumor cells with less toxicity and immunogenicity. Once invading into tumors, the bacteria spread throughout the whole tumor tissues and produce therapeutic agents to inhibit tumor cells (12). Additionally, other than the therapeutic agents, some bacteria capable of surviving in antigen-presenting cells (APCs) or utilizing type III secretion system (T3SS) can be engineered as vaccine vectors that deliver tumor antigens to APCs and induce durable tumor-specific CD8+ T cell responses (58, 59). To date, many bacterial species including *Clostridium sporogenes*, *Salmonella typhimurium*, *Bifidobacterium longum*, *E. coli*, *L. monocytogenes* and *P. aeruginosa* have been genetically modified as vectors for delivery of tumoricidal agents, immunomodulatory proteins or tumor antigens, which showed success in a variety of animal tumor models (60–65).

Gram-negative bacteria utilize T3SS to inject bacterial effectors into host cell cytoplasm (66). In the past decade, the delivery tools based on bacterial T3SS have attracted a lot of research attentions for development of therapeutic cancer vaccines (67). A French research group genetically modified the live attenuated *P. aeruginosa* strains as vaccine vectors that directly deliver tumor antigens to APCs *via* T3SS injection and trigger antigen-specific CD8+ T cell responses systemically, leading to long-lasting anti-tumor immune responses (59, 60, 68, 69). For instance, Epaulard et al. generated an attenuated *P. aeruginosa* strain CHA-OST S54-Ova with deletion of two T3SS toxins, exoenzyme S (ExoS) and ExoT, which were able to induce apoptosis, block production of reactive oxygen species and inhibit the phagocytic activity of host cells (70–73), and this *P. aeruginosa* strain was genetically modified to express a fusion gene encoding the N-terminal 54 amino acids of ExoS for T3SS-mediated translocation and the C-terminus of ovalbumin (OVA) for immunogenicity (60). Furthermore, the strain CHA-OST S54-Ova was able to elevate the number of OVA-specific CD8+ T cells *in vivo*, and the mice inoculated with CHA-OST S54-Ova were resistant to the challenge of OVA-expressing mouse melanoma cell line B16 (60). In a separated study, the authors developed another *P. aeruginosa* strain CHA-OAL by deleting four virulence genes, including *exoS*, *exoT*, *aroA* and *lasI*, which displayed reduced toxicity and enhanced efficiency for delivering tumor antigens (74). The *P. aeruginosa aroA* gene encodes an enzyme called 5-enolpyruvylshikimate 3-phosphate synthase, which is essential for synthesis of aromatic amino acids, and deletion of this gene was found to promote the intracellular growth of *P. aeruginosa* and elicit an increased level of opsonic antibodies in host against *P. aeruginosa* (75, 76). LasI is an acyl-homoserine lactone synthase that catalyzes the synthesis of N-(3-oxododecanoyl)-L-homoserine lactone (3O-C12-HSL), a quorum sensing signal molecule critical for regulating expression of many *P. aeruginosa* virulence factors, including ExoA, LasA protease, LasB elastase and alkaline protease (77, 78). Derouazi et al. engineered the *P. aeruginosa* CHA-OST to express a fusion protein comprising of the N-terminal 54 amino acids of ExoS and a tumor antigen TRP2 epitope, and identified that the TRP2 epitope (125-376) could activate the TRP2-specific CD8+ T cell response, leading to a significant protection of mice against glioma (69). In addition, a killed but metabolically active (KBMA) *P. aeruginosa* strain OSTAB was created by deletion of ExoS, ExoT and the two subunits of the exonuclease UvrABC, UvrA and UvrB, important for bacterial nucleotide excision repair, and it was subsequently photo-inactivated (79). This KBMA *P. aeruginosa* strain was incapable of replicating in host but still immunologically active with functional T3SS, which has been suggested to be a promising and safe antigen delivery vector for anti-tumor immunotherapy (68).

## *P. aeruginosa* ExoA-based Immunotoxins for Cancer Treatment

Bacterial toxins in cancer therapy have been extensively studied in the past decade, which effectively change the cellular functions and processes by influencing cell proliferation, differentiation and apoptosis, and eventually kill the tumor cells (57). The theory behinds the bacterial toxin-mediated cancer therapy is creation of chimeric proteins consisting of the catalytic part of a toxin responsible for killing tumor cells and a receptor-binding part such as an antibody or a receptor ligand for specific tumor targeting, and these chimeric proteins are termed as immunotoxins (80). Monoclonal antibodies are commonly used in generation of the tumor cell binding parts in immunotoxin, which bind to the specific molecules that are highly expressed on tumor cell membrane. Upon binding to the target molecules, the immunotoxins are endocytosed and released to host cytosol, ultimately inducing toxin-mediated cell death (81). Moreover, the antigen-binding domain of the antibody part in immunotoxins is usually shortened or modified to reduce immunogenicity (80). The most commonly used bacterial toxins for generation of immunotoxins include *Diphtheria* toxin and *Pseudomonas* exotoxin A (PE), which have showed great anti-tumor efficiency both *in vivo* and *in vitro*, and some of them are currently under clinical investigation (82, 83).

PE is the most toxic virulence factor in *P. aeruginosa* which inhibits protein synthesis through ADP-ribosylation of eukaryotic elongation factor 2 (84). It is a single polypeptide chain that can be divided into three functional domains, including receptor binding domain (I), translocation domain (II) and catalytic domain (III) (85). Moreover, the binding of PE receptor binding domain to the low density lipoprotein receptor related protein (LRP1), also known as CD91, on host cell surface mediates uptake of PE *via* receptor-mediated endocytosis (84). The PE-based immunotoxins were generated by replacing the PE receptor binding domain with the variable fragment (Fv) of a monoclonal antibody or a receptor ligand such as a growth factor or a cytokine, which targets a tumor-specific antigen or a receptor molecule overexpressed on tumor cell surface (86). The representative PE-based immunotoxins discovered and evaluated in the past decade for cancer treatment were summarized in **Table 1**.

**Table 1:** A summary of PE-based immunotoxins discovered and evaluated in the past decade for cancer treatment.

Immunotoxin names	Toxin part	Receptor-binding part	Target	Research type	Reference
HN3-ABD-T20	Truncated PE lacking domain II attached to ABD	Anti-GPC3 (HN3 nanobody)	Hepatocellular carcinoma cells	*In vitro* and *in vivo*	(87)
D7(VL-VH)-PE40	PE40	Anti-PSMA scFV	Prostate cancer cells	*In vitro* and *in vivo*	(88)
NZ-1-(scdsFv)-PE38KDEL	PE38KDEL	Anti-podoplanin (NZ-1) scdsFv	Malignant brain tumor cells	*In vitro* and *in vivo*	(89)
dhuVHH6-PE38	PE38	CD7 nanobody	T-cell acute lymphoblastic leukemia	*In vitro* and *in vivo*	(90)
2E4-PE38	PE38	Anti-CD25 scFv	Regulatory T cells	*In vivo*	(91)
HM1.24-ETA′	ETA′ (Truncated PE lacking domain I)	Anti-CD317 scFv	Myeloma cells	*In vitro* and *in vivo*	(92)
scFv13-ETA′	ETA′	Anti-CD13 scFv	Various cancer cells	*In vitro*	(93)
CPE−ETA’	ETA′	Claudin−4−binding domain of *Clostridium perfringens* enterotoxin	Various cancer cells	*In vitro*	(94)
D2C7-(scdsFv)-PE38KDEL	PE38KDEL	Anti-EGFR scdsFv	Glioblastoma	Phase I/II clinical trial	(95)
DARPin-LoPE	LoPE (Truncated PE lacking domain I, II and B cell epitopes)	HER2-specific DARPin	Ovarian cancer cells	*In vitro* and *in vivo*	(96)
EGF-PE40	PE40	EGF	Bladder cancer cells	*In vitro*	(97)
EGF-PE40, EGF-PE24mut	PE40 mPE24	EGF	Prostate cancer cells	*In vitro* and *in vivo*	(98)
CD89(scFv)-ETA′	ETA′	Anti-CD89 scFv	Myeloid leukemia cells	*In vitro*	(99)
HER2(scFv)-PE24	PE24	Anti-HER2 scFv	HER2-expressing breast cancer cells	*In vitro*	(100)
HER2-PE25-X7	PE25 with 7 point mutations in domain III	HER2-specific affibody molecule (ZHER2:2891)	HER2-expressing cancer cells	*In vitro* and *in vivo*	(101)
ADAPT_6_-ABD-PE25	PE25	ADAPT_6_	HER2-expressing breast cancer cells	*In vitro*	(102)
IL-4-PE	PE38KDEL	IL-4	Ovarian cancer cells	*In vitro* and *in vivo*	(103)
MSH-PE38KDEL	PE38KDEL	Melanophore-stimulating hormone	Melanoma cells	*In vitro* and *in vivo*	(104)
SS1(dsFv)PE38 (SS1P)	PE38	Anti-mesothelin scFv	Mesothelioma	Phase I clinical trial	(105)
J591scFvPE38QQR	PE38QQR	Anti-PSMA (J591) scFv	Prostate cancer cells	*In vitro* and *in vivo*	(106)
D7(VL-VH)-PE40	PE40	Anti-PSMA (D7) scFv	Prostate cancer cells	*In vitro*	(107)
BPC-Neu5Ac-Dimer-LL-ETA-RDEL	ETA′	Synthetic sialosides (BPC-Neu5Ac-dimers)	CD22-positive B-cell lymphoma cells	*In vitro*	(108)
VGRNb-PE	PE38	VEGFR2-specific Nanobody (3VGR19)	VEGFR2-expressing cancer cells	*In vitro*	(109)
hGC33−PE38	PE38	Anti-GPC3 (hGC33) scFv	Small cell lung cancer cells	*In vitro*	(110)
MOC31PE	PE	Anti- EpCAM (MOC31) scFv	Peritoneal surface malignancies	*In vitro* and *in vivo*	(111)
806-PE38	PE38	Anti-EGFR (m806) antibody scFv	Triple-negative breast cancer cells	*In vitro* and *in vivo*	(112)
scFv2A9-PE	PE38KDEL	Anti-EpCAM scFV	EpCAM-positive human hepatocellular carcinoma cells	*In vitro*	(113)
HN3-PE38	PE38	Anti-GPC3 (HN3) scFv	Hepatocellular carcinoma cells	*In vitro* and *in vivo*	(114)
LMB-12 LMB-100 LMB-164	PE domain III PE24 PE domain III attached to ABD	Anti-mesothelin** **scFv Anti-mesothelin Fab Anti-mesothelin** **scFv	Colorectal cancer cells	*In vitro* and *in vivo*	(115)
GD9P	PE38	GD9	CCK2R-expressing colorectal cancer cells	*In vitro* and *in vivo*	(116)
TGFα-PE38	PE38	TGFα	Various cancer cells	*In vitro* and *in vivo*	(117)
T22-PE24-H6	PE24	T22 (CXCR4 ligand)	CXCR4-positive diffuse large B-cell lymphoma cells	*In vitro* and *in vivo*	(118)

The naming of the truncated PE used in construction of the recombinant immunotoxins is usually based on their molecular weight. PE38 and PE40, two truncated forms of PE (38 kDa and 40 kDa, respectively), are most commonly used for immunotoxin construction, and both of them lack the receptor-binding domain (I) (119). Furthermore, as a foreign protein, the immunogenicity of immunotoxins is able to induce production of anti-drug antibodies in host, which neutralize and decrease the efficiency of the immunotoxins (82). Alteration of immunotoxin structure is a feasible strategy to reduce immunogenicity (120). Previous studies have reported that removal of the B cell or T cell epitopes from the PE-based immunotoxins could significantly reduce the immunogenicity and enhanced the anti-tumor efficiency both *in vitro* and *in vivo* (121–124). The B cell epitopes in PE38 were mapped by measuring the reactivity of PE38 to the monoclonal antibodies isolated from the mice or patients treated with the PE38-based immunotoxins (125), and they were removed by point mutations of the large hydrophilic amino acids such as arginine, glutamine, glutamic acid and lysine to alanine, serine or glycine, which prevents PE38 from binding to the B cell antigen receptors (124). The T cell epitopes were identified by incubating human peripheral blood mononuclear cells (PBMCs) or mouse splenocytes with whole PE38, and the reacting T-cells were subsequently stimulated with various PE38 peptides (122). Furthermore, the peptides capable of triggering T cell response were determined to contain the T cell epitopes, and the removal of T cell epitopes in PE38 could be achieved by deletion or point mutations (120). To date, many PE-based immunotoxins have been applied in clinical treatment of B-cell lymphoma (126), ovarian cancer (127), mesothelioma (105), breast cancer (128), esophageal cancer (128), brain cancer (95, 129, 130), and pancreatic adenocarcinoma (131). However, most of them are still in the early stage (phase I or II) of clinical trials, and the preliminary data indicated that toxicity and limited efficacy were the major challenges. In addition, the synergistic antitumor activity of PE-based immunotoxins combined with chemotherapeutic agents has been observed *in vitro* and in mouse tumor models (132–134). However, the combination of PE-based immunotoxins and chemotherapy lacks the clinical evidence for safe use in cancer patients. Alewine et al. demonstrated that the combination of immunotoxin LMB-100 and nab-paclitaxel could amplify the toxic side effects of LMB-100 (131).

## Conclusion and Future Perspectives

Tumor resistance to the conventional cancer therapies such as radiotherapy and chemotherapy is major cause of cancer relapse, and has led to a significant barrier in cancer treatment. The bacteria-based cancer therapy has emerged as a promising alternative or complementary strategy for cancer treatment, which exhibited great anti-tumor effects both *in vitro* and in animal tumor models. Among the therapeutic bacteria, *P. aeruginosa* takes advantages of large accessible genome, production of virulence factors with potent anti-tumor activities, and expression of various immunogenic molecules on membrane. The peritrichous *P. aeruginosa* strain PA-MSHA with MSHA fimbriae and low toxicity has been directly used as a therapeutic agent to destroy tumors by inducing tumor cell apoptosis, inhibiting tumor growth, and activating host immune responses. Moreover, the inactivated PA-MSHA combined with chemotherapy has proceeded to clinical trials. However, the PA-MSAH treatment seems to be ineffective to the patients who were tolerant to PA-MSHA stimulation. Therefore, a pre-test of the tolerance to PA-MSHA on cancer patients is recommended, and the patients with moderate adverse reactions will be proceeded for further treatment. The genetically engineered *P. aeruginosa* strain is able to activate tumor-specific CD8+ T cells by delivering tumor antigens to DCs, inducing long-lasting anti-tumor immunity. Moreover, for safety concern, the live attenuated *P. aeruginosa* strains should be unable to replicate, and are easily eliminated after treatment. PE is the most widely used *P. aeruginosa* toxin for construction of recombinant immunotoxins. Although the PE-based immunotoxins have shown significant *in vitro* and *in vivo* anti-tumor effects on nearly all types of tumors. However, only a few of them has proceeded to clinical practice, and the low efficiency and unanticipated toxicity to patients remain a big challenge that must be overcome in clinical applications. In future, development of new PE-based immunotoxins with high specificity and less immunogenicity should be one of the major tasks in bacteria-based cancer therapy, which is challenging but rewarding. Overall, the *P. aeruginosa*-based cancer therapies are promising strategies for cancer treatment, and they are particularly more effective in combination with conventional cancer therapies.

## Author Contributions

ZP contributed to the conceptualization, manuscript writing, supervision and funding acquisition. M-DG contributed to manuscript writing. TT made the figures and edited the manuscript. All the authors read and approved the final manuscript.

## Funding

This study was funded by the National Natural Science Foundation of China (Grant No. 82002112).

## Conflict of Interest

The authors declare that the research was conducted in the absence of any commercial or financial relationships that could be construed as a potential conflict of interest.

## Publisher’s Note

All claims expressed in this article are solely those of the authors and do not necessarily represent those of their affiliated organizations, or those of the publisher, the editors and the reviewers. Any product that may be evaluated in this article, or claim that may be made by its manufacturer, is not guaranteed or endorsed by the publisher.

## Abbreviations

ExoA, exotoxin A; CARD, caspase recruitment domain; PA-MSHA, *Pseudomonas aeruginosa*-mannose sensitive hemagglutinin; MSHA, mannose-sensitive hemagglutination; TLR4, toll-like receptor 4; IRAEs, immune-related adverse events; ER, endoplasmic reticulum; APC, antigen-presenting cell; T3SS, type III secretion system; ExoS, exoenzyme S; ExoT, exoenzyme T; OVA, ovalbumin; 3O-C12-HSL, N-(3-oxododecanoyl)-L-homoserine lactone; KBMA, killed but metabolically active; LRP1, low density lipoprotein receptor related protein; PE, *Pseudomonas* exotoxin A; Fv, variable fragment; PBMC, peripheral blood mononuclear cell.
